# Atomoxetine effects on attentional bias to drug-related cues in cocaine dependent individuals

**DOI:** 10.1007/s00213-017-4643-4

**Published:** 2017-05-27

**Authors:** Luca Passamonti, M. Luijten, H. Ziauddeen, I. T. S. Coyle-Gilchrist, T. Rittman, S. A. E. Brain, R. Regenthal, I. H. A. Franken, B. J. Sahakian, E. T. Bullmore, T. W. Robbins, K. D. Ersche

**Affiliations:** 1 Departments of Clinical Neurosciences, University of Cambridge, Cambridge, CB2 0SZ UK; 2 Consiglio Nazionale delle Ricerche, Istituto di Bioimmagini e Fisiologia Molecolare, Catanzaro, Italy; 3 Behavioural Science Institute, Radboud University, Nijmegen, Netherlands; 4 Cambridgeshire and Peterborough Foundation Trust, Cambridge, UK; 5 Division of Clinical Pharmacology, Rudolf-Boehm-Institute of Pharmacology and Toxicology, University of Leipzig, Leipzig, Germany; 6 Institute of Psychology, Erasmus University Rotterdam, Rotterdam, Netherlands; 7 Behavioural and Clinical Neuroscience Institute, University of Cambridge, Cambridge, UK; 8 GlaxoSmithKline, Clinical Unit Cambridge, Cambridge, UK

**Keywords:** Attentional bias, Response inhibition, Cocaine addiction, Noradrenaline, Atomoxetine

## Abstract

**Rationale:**

Biased attention towards drug-related cues and reduced inhibitory control over the regulation of drug-intake characterize drug addiction. The noradrenaline system has been critically implicated in both attentional and response inhibitory processes and is directly affected by drugs such as cocaine.

**Objectives:**

We examined the potentially beneficial effects of the noradrenaline reuptake inhibitor atomoxetine in improving cognitive control during two tasks that used cocaine- and non-cocaine-related stimuli.

**Methods:**

A double-blind, placebo-controlled, and cross-over psycho-pharmacological design was employed. A single oral dose of atomoxetine (40 mg) was administered to 28 cocaine-dependent individuals (CDIs) and 28 healthy controls. All participants performed a pictorial attentional bias task involving both cocaine- and non-cocaine-related pictures as well as a verbal go/no-go task composed of cocaine- and food-related words.

**Results:**

As expected, CDIs showed attentional bias to cocaine-related cues whilst controls did not. More importantly, however, atomoxetine, relative to placebo, significantly attenuated attentional bias in CDIs (*F*_26_ = 6.73, *P* = 0.01). During the go/no-go task, there was a treatment × trial × group interaction, although this finding only showed a trend towards statistical significance (*F*_26_ = 3.38, *P* = 0.07).

**Conclusions:**

Our findings suggest that atomoxetine reduces attentional bias to drug-related cues in CDIs. This may result from atomoxetine’s modulation of the balance between tonic/phasic activity in the locus coeruleus and the possibly parallel enhancement of noradrenergic neurotransmission within the prefrontal cortex. Studying how cognitive enhancers such as atomoxetine influence key neurocognitive indices in cocaine addiction may help to develop reliable biomarkers for patient stratification in future clinical trials.

## Introduction

Addiction to cocaine remains a significant public health problem, and with current treatment provision confined to psychosocial interventions, there is a pressing need to improve the breadth and nature of the therapeutic approaches to cocaine dependence (Goldstein et al. 2009; Lapeyre-Mestre and Dupui 2015; Wiessing 2005). There is also a consensus that enhancing our knowledge of the cognitive factors that underlie cocaine taking might help in developing new interventions to ameliorate the chances of recovery in cocaine-dependent individuals (CDIs).

Cocaine-related cues capture the attention of CDIs and may influence their cocaine-taking behaviour (Marissen et al. 2006; Waters et al. 2012). Whilst performing cognitive tasks, CDIs, relative to controls, display biased attention towards distracting cocaine-related stimuli (Dunning et al. 2011; Ersche et al. 2010; Field et al. 2014; Goldstein et al. 2007; Hester et al. 2006; Leeman et al. 2014; Smith et al. 2013), although this ‘attentional bias’ should not necessarily be considered to be abnormal per se as it can also be observed in healthy people even in the absence of any experimental manipulation (Pintzinger et al. 2016) or can be trained towards stimuli linked to salient and evolutionary important outcomes (Schmidt et al. 2016).

The inhibitory control of maladaptive impulsive responses is another key mechanism that influences cocaine-taking behaviour; in other words, impulsivity represents a critical predictor of vulnerability for cocaine taking and relapse (Bosker et al. 2017; Ersche et al. 2012; Goldstein and Volkow 2002; Robbins et al. 2012). Although attentional bias and response disinhibition are distinct phenomena, attentional bias can clearly be influenced by cognitive control mechanisms including response inhibition (Bari and Robbins 2013). In particular, inhibitory mechanisms are fundamental in attentional tasks for limiting the amount of information accessible at any time and filtering out distracting environmental cues that may degrade behavioural performance (Bari and Robbins 2013).

It follows that attenuating attentional bias to cocaine-related cues and potentiating response inhibition, via psychotherapy and/or pharmacotherapy, might help the development of new treatments to reduce cocaine-taking behaviour (Marhe et al. 2014). One potential approach could utilize pharmacological interventions to enable CDIs to recruit greater cognitive control to reduce attentional bias to cocaine-related cues and ameliorate response inhibition. An excellent candidate for this approach is atomoxetine, which inhibits the reuptake of noradrenaline in the synaptic cleft by blocking the noradrenaline transporter (Kratochvil et al. 2003). There is accumulating evidence that dysfunctioning noradrenergic transmission may be important in cocaine addiction (Schmidt and Weinshenker 2014), consistent with data from animal research showing that atomoxetine may attenuate the risk for relapse following cocaine abstinence (Economidou et al. 2011). However, a clinical trial that investigated the efficacy of atomoxetine in reducing cocaine use in CDIs was less promising than initial comparative studies (Walsh et al. 2013). Nevertheless, these are not necessarily conflicting findings, as drug taking and drug relapse have been shown to be separate processes that may involve distinct neural circuitry and, in many cases, can be differentially affected by pharmacotherapy. For example, both clinical and pre-clinical studies have shown that NET inhibitors may not be effective in decreasing ongoing drug use whilst other adrenergic agents (e.g. alpha 2 agonists) may reduce drug relapse (Fox and Sinha 2014; Fox et al. 2015; Smith and Aston-Jones 2011).

Investigating how atomoxetine influences key neurocognitive markers such as attentional bias to cocaine-related cues and response inhibition in CDIs has therefore the potential to advance from purely descriptive accounts of behavioural problems to predictive and mechanistic models with the prospective for novel, rational, and individualized therapies. The main aim of this study was to test the hypothesis that a single dose of atomoxetine, relative to placebo, attenuates attentional bias to cocaine-related cues and potentiates response inhibition in CDIs. We employed two well-validated cognitive tasks that consistently index attentional bias and response inhibition (Chamberlain et al. 2009; Luijten et al. 2011; Noel et al. 2005). To elicit attentional bias to cocaine-related cues in CDIs, we adapted a task in which participants have to count the number of lines superimposed over distracting cocaine-related pictures (e.g. people smoking crack) (Luijten et al. 2011). The main advantage of this paradigm is that cocaine-related photographs evoke stronger reactions (thereby amplifying the attentional bias) in CDIs compared to the word stimuli typically used in Stroop tasks (Boyer and Dickerson 2003; Cane et al. 2009; Cox et al. 2006; Ryan 2002).

Impulsivity and inhibitory control were assessed using a go/no-go task which included cocaine-related words as the main stimuli and control stimuli with motivational/appetitive salience (i.e. food-related words) (Noel et al. 2005). Atomoxetine (40 mg) has been found to potentiate response inhibition in healthy individuals (Chamberlain et al. 2006). Whilst some studies have failed to replicate this effect (Nandam et al. 2011) or have found opposite results (Graf et al. 2011), this might have depended critically on the dose of atomoxetine; in the present study, we employed the optimal dose (40 mg) as used previously (Chamberlain et al. 2006).

Finally, we explored whether and how clinical and personality variables (i.e. years of cocaine abuse, dysphoric mood, trait impulsivity) influenced the cognitive enhancing effects of atomoxetine (i.e. decreased attentional bias to cocaine-related cues and enhanced inhibitory control). Although we did not formally assess a possible diagnosis of attention-deficit/hyperactivity disorder (ADHD) using the DSM-IV criteria, the CDI individuals completed the adult ADHD Self-Report Scale (ASRS), which is a measure of individual differences in ADHD symptoms (Kessler et al. 2005). This is because previous evidence showed that atomoxetine may be useful in CDIs with ADHD symptoms (Levin et al. 2009).

## Participants and methods

### Participants

The study was approved by the National Research Ethics Committee and all participants provided written informed consent. Twenty-eight individuals who met the DSM-IV-TR criteria for cocaine dependence and 28 age- and gender-matched healthy controls with no history of dependence or other neuropsychiatric disorders took part in the study. Individuals with CDI were recruited via local treatment services and word of mouth. The healthy volunteers were recruited from the Cambridge BioResource volunteer panel (www.cambridgebioresource.org.uk).

All participants were screened for medical and neuropsychiatric disorders by medical history, laboratory testing, physical examination, and electrocardiogram. Exclusion criteria for all participants were as follows: (1) any current or past serious medical illness such as hepatic, cardiac, renal, infectious, metabolic, or pulmonary disease; (2) any history of traumatic head injury; (3) pregnancy; (4) no proficiency in English; and (5) any involvement in a clinical trial investigating drug effects within the past 6 months. For controls, a personal history of any psychiatric disorder led to exclusion whilst for the cocaine group, a personal history of a psychotic disorder led to exclusion.

All participants were screened for current psychiatric disorders using the Mini-International Neuropsychiatric Inventory (MINI) (Sheehan et al. 1998), and drug use was further evaluated using the Structured Clinical Interview for DSM-IV (SCID) (First 2002). Participants also completed the National Adult Reading Test (NART) to assess verbal intelligence quotient (IQ), the Beck Depression Inventory (BDI-II) to record dysphoric mood, the Spielberger State-Trait Anxiety Inventory (STAI) to evaluate anxiety levels, and the Barratt Impulsiveness Scale (BIS-11) to assess trait impulsivity. CDIs completed the obsessive-compulsive drug use scale (Franken et al. 2002) and the ASRS scale to assess for individual differences in ADHD symptoms (Kessler et al. 2005). Last, self-reported feelings of cocaine craving in CDIs were collected immediately after each testing session (i.e. placebo and atomoxetine).

The attentional bias task to cocaine-related cues and go/no-go task were conducted at the Addenbrooke’s Hospital in Cambridge (UK) in two separate sessions following a double-blinded design with a counterbalanced order across participants and treatment (i.e. atomoxetine, placebo). On each session, urine samples were analysed for cocaine. All the urine samples provided by CDIs tested positive for cocaine and all of the urine samples provided by the controls tested cocaine negative. The absence of acute alcohol intoxication was verified using breath tests. At the beginning of each session, a single dose of 40 mg atomoxetine or placebo was given orally and vital signs were measured. Participants were dosed either at 11.00 am or at 12.00 pm. After approximately 2 h, vital signs were measured again and blood samples taken to assess atomoxetine plasma concentrations. Finally, approximately 2.5 h after atomoxetine dosing, all volunteers performed the cognitive tests. This schedule was based on pharmacokinetics data declared in the Strattera® (atomoxetine hydrochloride) leaflet which reports that plasma concentration of atomoxetine peaks approximately 2 h after a single oral dose of 40 mg and remains at ~60% peak up to 6 h.

### Experimental tests

#### Line-counting task with cocaine-related and neutral pictures

We employed a modified version of the paradigm originally developed by Luijten et al. (2011), in which participants were asked to indicate, via a button-press response, the number of lines superimposed on photographic images displayed on the screen. Seventy-two coloured photographs, half depicting images of non-cocaine-related objects and activities (household items; individual handling household items) and the other half showing cocaine-related objects and activities (a crack pipe; individual smoking a crack pipe), were presented twice to participants. On each photograph, semi-randomly spaced blue horizontal lines were superimposed. The number of lines varied between 2 and 5, and participants had to respond by pressing the corresponding number keys on the keyboard. The images were matched across the two conditions (neutral, cocaine) for colour, brightness, object size, object position, and the number of blue lines laid over them.

The task was administered in eight runs, each consisting of a block of nine neutral images and nine cocaine-related photographs. The order of the category to be shown first within a block (neutral or cocaine) was randomized to control for order effects. Each photograph was displayed in the centre of the computer screen for 900 msec and participants were asked to respond as soon as possible when the picture appeared on the screen. The inter-stimulus interval was jittered across trials and on average lasted 1750 msec. All participants were trained on the task with a practice run of 30 unrelated colour photographs (depicting animals, people, or landscapes) to familiarize themselves with the task and the response buttons.

Attentional bias to cocaine-related pictures was quantified by an interference score, which was calculated as the mean latency of correct responses to the cocaine-related images minus the mean latency to correct responses to the non-cocaine-related images.

#### Go/no-go task with cocaine-related and food-related words

A modified version of a verbal go/no-go task previously developed by Noel et al. (2005) was employed. The task included cocaine-related words and food-related words, each of which was displayed, one by one, in the middle of the screen. Half of the words were targets to which participants were instructed (via a visual cue) to respond as quickly as possible by pressing a button, and half of them were distracters to which participants had to refrain responding. Each word was presented for 500 msec, with an inter-stimulus interval of 900 msec between two words.

Eighteen word stimuli (nine cocaine-related words and nine food-related words) were grouped in a block, and within each block, either cocaine- or food-related words were specified as targets. The cocaine- and food-related words were as closely matched as possible in terms of word length and frequency. Examples of cocaine-related words were ‘amphetamine’, ‘crack’, and ‘dealer’, whilst words like ‘marshmallows’, ‘pizza’, and ‘cherry’ were used as food-related words. Whether participants started with cocaine-related or food-related words was randomized within each group. Reaction times (RTs) to respond to a target were calculated for all participants and RTs <100 msec (anticipated responses) were excluded from the analyses.

As in a previous study (Sahgal 1987), we computed the number of false alarms (i.e. response to a distracter) and hits (correct response to a target) that were both used to calculate a response bias score. The response bias score was calculated as follows: response bias = −0.5 × (*Z* (corrected probability of hits) + *Z* (corrected probability of false alarms)), where *Z* scores were calculated using the inverse phi function, which determines the *Z* score of *P* values. The corrected probability of hits was also computed as follows: corrected probability of hits = (number of correct go responses +0.5)/(total number of go trials +1) and the corrected probability of false alarms = (number of incorrect no-go responses +0.5)/(total number of no-go trials +1).

Given that the response bias score takes into account both correct responses and false alarms, it can be considered a better indicator of impulsivity and disinhibition than false alarms alone (Sahgal 1987). A more negative response bias score reflects higher impulsivity (Noel et al. 2005).

### Plasma analysis

Plasma levels of atomoxetine were analysed in all participants, with a high-performance liquid chromatographic method (Agilent Technologies GmbH, Waldbronn, Germany). Separation of atomoxetine and mianserine (internal standard) was performed on an Agilent Zorbax Eclipse XDB C8 reversed phase column. Detection wavelength was 218 nm, and the limit of quantitation was 2.0 μg/l. Importantly, there were no any additional peaks in the samples indicative of atomoxetine metabolites.

### Statistical analyses

Data were analysed using the SPSS (v.24) software. Independent two sample *t* tests were used for group comparisons on demographic, cognitive, and personality variables as well as vital signs (i.e. pulse, systolic, and diastolic blood pressure).

Furthermore, the interference scores for the line-counting task and the response bias scores for the cocaine-related go/no-go task were entered in repeated-measure general linear models (GLM) that tested for (1) the main effect of group, (2) main effect of treatment, (3) main effect of stimulus (for the go/no-go task), and (4) any group × treatment interaction or group × stimulus and group × treatment × stimulus interaction (the latter two only for the go/no-go task). The main effect of and interactions involving the stimulus (cocaine, food) were only possible for the drug-related go/no-go task as the interference scores for the attentional bias task were calculated in terms of reaction times to cocaine-related minus neutral stimuli.

Individual plasma levels of atomoxetine were correlated (using the Pearson’s correlation coefficient) with (1) the difference in the interference measures during the attentional bias task (calculated by subtracting the interference scores during placebo from the same metrics under atomoxetine) and (2) the difference in the response bias scores during the cocaine-related go/no-go task (computed by subtracting the response bias indices during placebo from the same metrics under atomoxetine). Using repeated-measure GLM, we also assessed whether self-reported feelings of cocaine craving changed after each tasks and whether they were influenced by atomoxetine.

Finally, the differences in the interference scores between atomoxetine and placebo conditions for the line-counting task as well as the differences for the response bias scores for the cocaine-related go/no-go paradigm were correlated with the BDI-II, BIS-11, and ASRS scores as well as with the number of years of cocaine use. All statistical tests performed were two-tailed and the significance level threshold was set at *P* < 0.05.

## Results

### Demographics, cognitive, and personality data

The groups were well-matched in terms of gender and age (Table 1). Furthermore, there were no differences between groups in pulse rate, systolic, or diastolic blood pressure suggesting that cocaine users were not acutely intoxicated (Table 1). CDIs scored significantly higher on the BDI-II and BIS-11 questionnaires compared to controls (Table 1). Self-reported feelings of cocaine craving did not increase significantly after the task (*F*1,25 = 1.83, *P* = 0.18) and were not affected by atomoxetine (*F*1,25 = 0.59, *P* = 0.44).

**Table 1:** Demographics, cognitive, personality, and baseline characteristics of the sample

	Controls (*n* = 28)	CDIs (*n* = 28)	Group differences
Age (years ± SD)	44.7 ± 7.4	41.1 ± 7.4	*F* = 0.14, *P* = 0.70
Gender (males/females)	26/2	27/1	NS
Education (years ± SD)	12.8 ± 2.8	11.5 ± 1.8	*F* = 4.7, *P* = 0.03
Verbal IQ, NART (mean scores ± SD)	115.2 ± 6.7	102.3 ± 8.4	*F* = 0.94, *P* = 0.33
Dysphoric mood, BDI-II (mean score ± SD)	3.0 ± 4.3	16.0 ± 8.6	*F* = 13.2, *P* = 0.001
Trait impulsivity. BIS-11 (mean score ± SD)	58.4 ± 6.8	72.9 ± 9.9	*F* = 5.3, *P* = 0.025
Trait anxiety, STAI (mean score ± SD)	29.1 ± 7.2	41.7 ± 8.4	*F* = 36.7, *P* < 0.001
Adult ADHD self-report scale (ASRS)	40.7 ± 8.3	48.4 ± 9.5	*T* = 3.2, *P* = 0.002
Pulse rate (ppm ± SD)	67.3 ± 11.8	70.6 ± 12.0	*F* = 0.003, *P* = 0.96
Systolic blood pressure (mmHg ± SD)	121.2 ± 11.5	120.9 ± 14.3	*F* = 1.1, *P* = 0.29
Diastolic blood pressure (mmHg ± SD)	73.6 ± 8.9	73.2 ± 9.8	*F* = 0.26, *P* = 0.60
Discrimination data (*D*′) during the go/no-go task for the drug word stimuli (on atomoxetine)	2.7 ± 0.8	1.8 ± 1.0	*T* = 3.4, *P* = 0.001
Discrimination data (*D*′) during the go/no-go task for the drug word stimuli (under placebo)	2.7 ± 0.8	1.8 ± 0.8	*T* = 3.9, *P* < 0.001
Discrimination data (*D*′) during the go/no-go task for the food word stimuli (on atomoxetine)	3.0 ± 0.7	2.2 ± 0.8	*T* = 3.9, *P* < 0.001
Discrimination data (*D*′) during the go/no-go task for the word stimuli (under placebo)	2.9 ± 0.7	1.9 ± 0.8	*T* = 4.6, *P* < 0.001

The discrimination data during the go/no-go task were calculated using a signal detection analysis (*D*′) (Snodgrass and Corwin 1988). *D*′ < or = 0 indicates that participants were either unable to discriminate targets from distracters or they were not performing the task as instructed. A *D*′ > 0 reflects good discrimination ability (e.g. more hits and less false alarms)

*SD* standard deviation, *CDIs* cocaine-dependent individuals, *IQ* intelligence quotient, *NART* National Adult Reading Test, *BDI-II* Beck Depression Inventory-second edition, *BIS-11* Barratt Impulsiveness Scale-11, *STAI* Spielberger State-Trait Inventory, *ppm* pulse per minute, *NS* not significant by means of a *χ* ^2^ test

### Behavioural performance

#### Attentional bias during the line-counting task with cocaine-related and neutral pictures

**Fig. 1:**
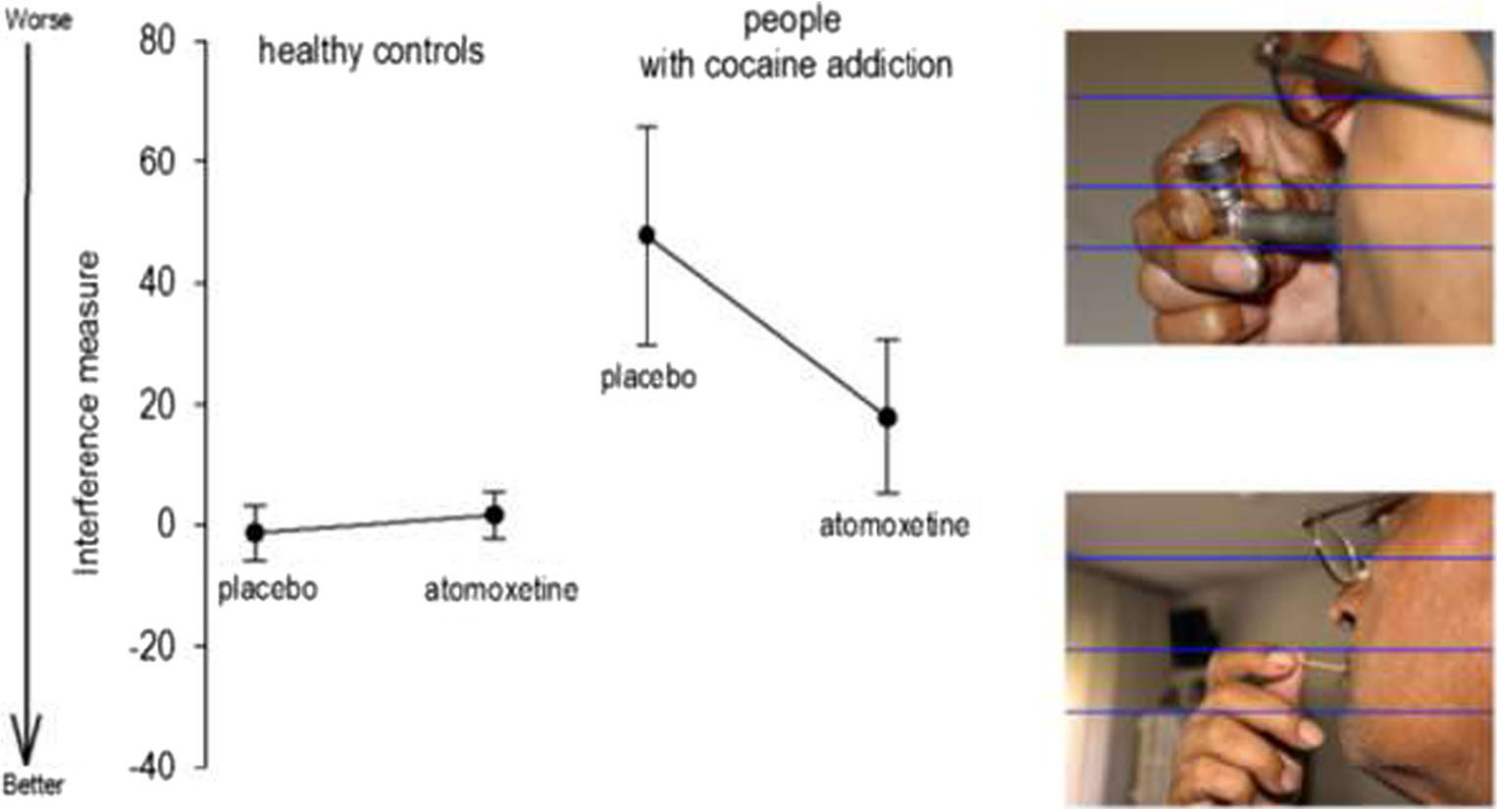
This figure displays the differences between groups and treatment conditions in the interference measure (an index of attentional bias) that was calculated by subtracting the mean response latency during non-drug-related conditions (*bottom right panel* for an example) from the mean response latency during drug-related pictures presentation (*top right panel* for an example). Overall, there was a significant main effect of group that was driven by a generally increased attentional bias to drug-related cues in cocaine-dependent individuals relative to controls. More importantly, however, there was a significant group by treatment interaction that was dependent on the fact that atomoxetine administration reduced the attentional bias to drug-related pictures selectively in the cocaine group. *Black circles* represent the mean values per each group and treatment condition whilst the *capped lines* denote the standard errors

There was a significant main effect of group with increased interference scores in CDIs relative to controls, reflecting increased attentional bias to cocaine-related photographs in CDIs (*F*26 = 5.34, *P* = 0.02) (Fig. 1). Whilst there was no significant main effect of treatment (*F*26 = 2.42, *P* = 0.13), there was a significant group × treatment interaction (*F*26 = 6.73, *P* = 0.01). Specifically, post hoc analyses showed that atomoxetine reduced the attentional bias to cocaine-related pictures in CDIs but there was no difference between atomoxetine and placebo in controls (Fig. 1).

#### Response inhibition during the go/no-go task with cocaine- and food-related words

**Fig. 2:**
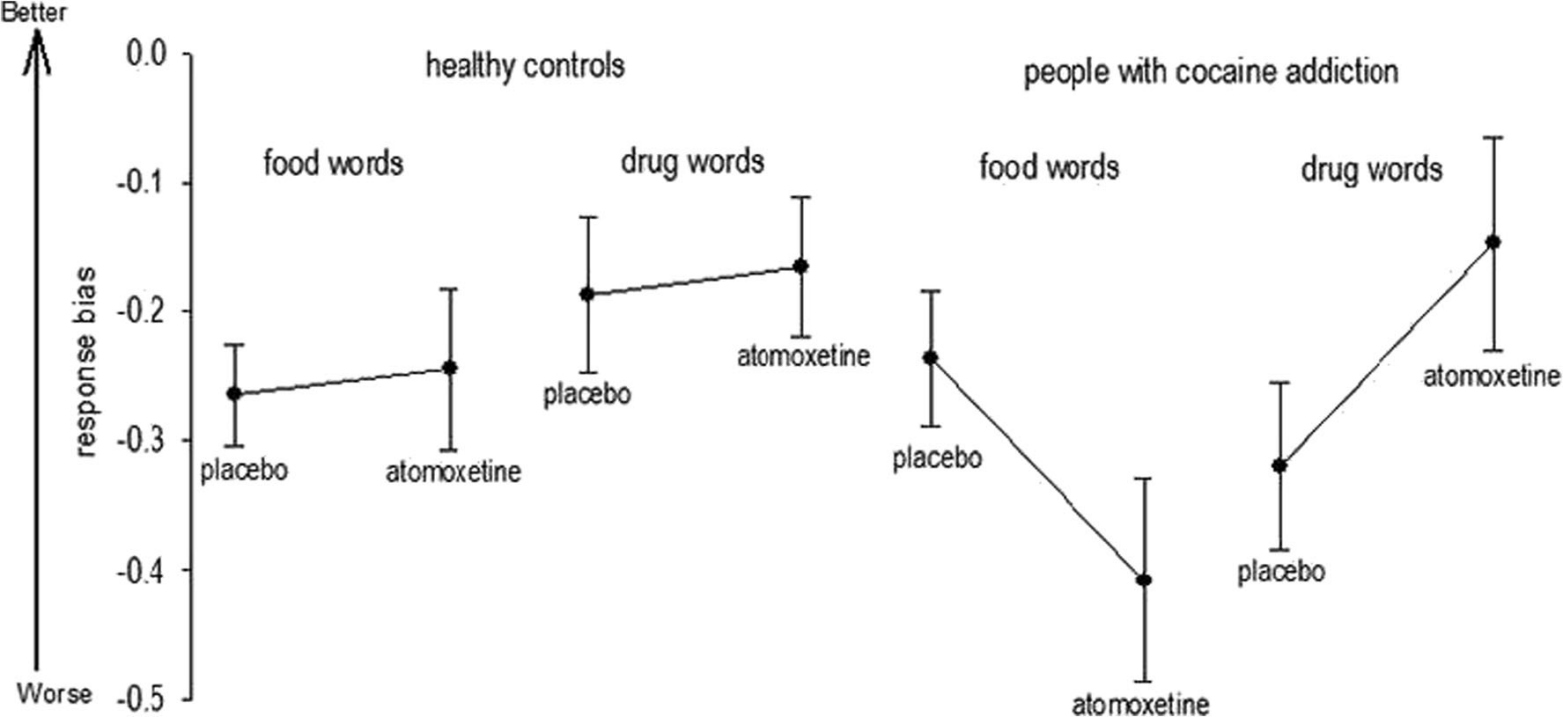
The plot displays the differences between groups, stimulus type, and treatment conditions in the response bias score (an indicator of impulsivity). Overall, atomoxetine reduced impulsive responding for drug-related target words and had the opposite effect when food-related words were the target, particularly in people with cocaine addiction

There was no main effect of group (*F*26 = 0.64, *P* = 0.42) or treatment (*F*26 = 0.08, *P* = 0.77), although we found a main effect of stimulus type which was driven by increased response bias to cocaine relative to food-related words (*F*26 = 6.37, *P* = 0.01) (Fig. 2). There was no significant group × treatment interaction (*F*26 = 0.15, *P* = 0.69), although a trend towards statistical significance for the group × stimulus × treatment interaction was found (*F*26 = 3.38, *P* = 0.07). A post hoc comparison between groups during the placebo condition was not statistically different (*T* = 1.15, *P* = 0.13) indicating that CDIs were not more impulsive than controls on baseline.

### Correlational analyses

No significant correlations were found between plasma atomoxetine levels and (1) the difference between interference scores under atomoxetine and the same measures under placebo for the attentional bias task and (2) the differences between response bias scores under atomoxetine and the same measures under placebo for the cocaine-related go/no-go task (Pearson’s *r*’s < 0.19, *P*’s > 0.18 or *r*’s > −0.21, *P*’s > 0.11).

On examining the relationship between the attentional bias to cocaine-related cues, response inhibition measures, and clinical as well as personality variables (i.e. years of cocaine use, BDI-II, BIS-11, and ASRS scores), we found that the difference in attentional bias measures between atomoxetine and placebo conditions was only (and positively) correlated with the years of cocaine use (Person’s *r* = 0.48, *P* = 0.02). In other words, CDIs with the longest history of cocaine use gained the least benefit from atomoxetine in terms of reducing attentional bias to cocaine-related cues. However, excluding an individual with 5 years of cocaine use made this result of borderline significance (*r* = −.38, *P* = 0.08), so we no further discuss it.

## Discussion

We provide new evidence that atomoxetine significantly decreased attentional bias to cocaine-related stimuli in cocaine-dependent individuals (CDIs). Overall, these findings show that atomoxetine impacts on a key neurocognitive measure in cocaine addiction—i.e. attentional bias to cocaine-related cues (Marhe et al. 2013; Marissen et al. 2006).

Although the molecular mechanisms of the cognitive enhancing properties of atomoxetine require further investigation, a previous animal study has demonstrated that atomoxetine diminishes tonic firing of the locus coeruleus (LC) whilst leaving phasic bursting intact (Bari and Aston-Jones 2013). Together, these mechanisms are in keeping with the traditional theory of LC noradrenergic function which posits that performance on cognitive tasks is optimal at intermediate levels of tonic noradrenergic transmission but deteriorates when too little or too much noradrenaline is available (Aston-Jones et al. 1999; Bari and Aston-Jones 2013). In the context of our data, we speculate that atomoxetine may alter the balance between the tonic and phasic LC firing which overall reduces distractibility and attentional bias to cocaine-related cues in CDIs (Bari and Aston-Jones 2013). Conversely, atomoxetine did not influence task performance in controls as these persons already display an optimal range of noradrenaline transmission, and perhaps more importantly, as expected, they did not show attentional bias to cocaine-related cues. Consequently, it remains to be tested whether atomoxetine can also modulate other types of attentional bias in healthy individuals.

In terms of likely terminal domains for the effects of atomoxetine, the drug increases noradrenaline levels in the prefrontal cortex (PFC) which in turn would enhance ‘top-down’ cognitive control (Arnsten 2009). Atomoxetine has also been found to increase extracellular levels of dopamine as well as noradrenaline in the PFC (Bymaster et al. 2002) and so it is also possible that the present effects were mediated by PFC dopamine. However, a previous study reported that the effects of atomoxetine on response inhibitory control in rats were mediated by noradrenergic rather than dopaminergic neurotransmission (Bari et al. 2011). A non-mutually exclusive possibility is that atomoxetine simultaneously affects noradrenergic- and dopaminergic-related mechanisms which in turn act in concert to modulate specific PFC circuits mediating attentional control (Briand et al. 2007).

In addition, we found a trend towards statistical significance (*P* = 0.07) for the group × stimulus × treatment interaction during the go/no-go task. A post hoc comparison between groups during the placebo condition was not statistically different (*T* = 1.15, *P* = 0.13) indicating that CDIs were not more impulsive than controls on baseline. However, this result may warrant a replication with larger samples.

Our findings suggest new avenues for future research, for example, to elucidate the brain mechanisms underlying the atomoxetine effects on attentional bias to cocaine-related cues using functional magnetic resonance imaging (fMRI). A previous fMRI study found that smokers, relative to non-smokers, showed greater dorsal anterior cingulate cortex (dACC) activity whilst attending to task-relevant information in the presence of distracting smoke-related cues (Luijten et al. 2011). This indicates that smokers excessively recruit the dACC to achieve the necessary attentional control for task performance. Increased dACC activation during a Stroop paradigm has also been found to predict relapse in cocaine use 3 months after detoxification (Marhe et al. 2013). Together, these results suggest that task-related brain function in specific regions could be a promising biomarker in people with substance dependence, although it remains to be elucidated whether it can be used to identify those CDIs most likely to benefit from pharmacological interventions such as atomoxetine.

Although a previous clinical trial provided no support for the use of atomoxetine in treating cocaine dependence (Walsh et al. 2013), this null finding may have in part been due to the lack of reliable neurocognitive indices to stratify patients and to the use of pharmacological treatments which were limited in time. Hence, studies assessing the effects of chronic atomoxetine therapy to evaluate long-term changes in neurocognitive and behavioural outcome measures in selected groups of CDIs are warranted.
